# Shallow water marine gammaridean amphipods of Pulau Tioman, Malaysia, with the description of a new species

**DOI:** 10.3897/zookeys.335.5567

**Published:** 2013-09-24

**Authors:** B.A.R. Azman, B.H.R. Othman

**Affiliations:** 1 Marine Ecosystem Research Centre (EKOMAR), Faculty of Science and Technology, Universiti Kebangsaan Malaysia, 43600 UKM Bangi, Selangor, Malaysia

**Keywords:** South China Sea, Amphipoda, Gammaridea, taxonomy, new species, *Tethygeneia sunda*

## Abstract

Eleven taxa including one new species of gammaridean amphipods are reported from the waters of Pulau Tioman. The presence of *Tethygeneia sunda* **sp. n.** represents the first record of the genus from the South China Sea. Additional material of *Ampelisca brevicornis* (Costa, 1853); *Cymadusa vadosa* Imbach, 1967; *Paradexamine setigera* Hirayama, 1984; *Ericthonius pugnax* (Dana, 1853); *Leucothoe furina* (Savigny, 1816); *Microlysias xenokeras* (Stebbing, 1918); *Monoculodes muwoni* Jo, 1990 are identified from the South China Sea, supporting previous records by Lowry (2000), Huang (1994), Imbach (1967), Margulis (1968) and Nagata (1959). Three additional species, *Gitanopsis pusilla* K.H. Barnard, 1916, *Liljeborgia japonica* Nagata, 1965b and *Latigammaropsis atlantica* (Stebbing, 1888), whilst previously reported from the neighbouring waters, comprise new records for the South China Sea.

## Introduction

According to Lim et al. (2010), taxonomic knowledge on the gammaridean amphipods from the waters of Peninsular Malaysia has been poorly studied in the past. Whilst there have been several revisions of species or even genera around Malaysia, most of the studies have been of a sporadic nature, with miscellaneous small papers on various taxa (e.g Muller 1993; Othman and Morino 1996, 2006; Othman and Azman 2007; Tomikawa et al. 2007; Azman and Melvin 2011, and Azman and Othman 2012). All these indicate the regional gammaridean taxa are largely poorly known but likely to be diverse and potential diversity of species still waiting for our investigation.

Particular efforts in conducting more regional based studies should be emphasized to further advance the biodiversity knowledge of these numerically abundant and taxonomically diverse taxa. Hence, the result of this work should in addition to documenting the amphipod fauna, provide new insights into the diversity and distribution patterns of the South China Sea amphipods.

Located 2°35’ north of the equator and in the South China Sea, Pulau Tioman (*pulau* = island) is an island of approximately 100 km^2^ in area, lying 20 km off the southeastern coast of the Malay Peninsular. Apart from its beautiful beaches and marine ecosystems, the marine areas around Pulau Tioman and eight other nearby islands (Pulau Tulai, Pulau Sepoi, Pulau Chebeh, Pulau Tokong Bahara, Pulau Sembilang, Pulau Sri Bulat, Pulau Labas and Pulau Gut) have also been gazzeted as marine parks and marine reserves under the Fisheries Act (1985). The establishment of the marine parks and marine reserves is to conserve, protect marine fauna and flora such as fishes, coral reefs and aquatic floras from being destroyed by fishing and other human activities.

In the framework of research on taxonomic revision and ecology of selected families of gammaridean amphipods conducted at Pulau Tioman, several qualitative benthic samples from different habitats and substrate types have been analysed. In some of these samples, specimens of gammaridean amphipods were found, one of which belongs to new species herein described, and for those species already known, the morphology of populations from Pulau Tioman are compared with other populations described in the literature. Due to the high importance of Pulau Tioman to maintain inventories for scientific marine studies in the Marine Protected Areas of Malaysia, we consider it important to include these species in this report, together with the description of the new taxa.

## Material and methods

**Figure 1.**
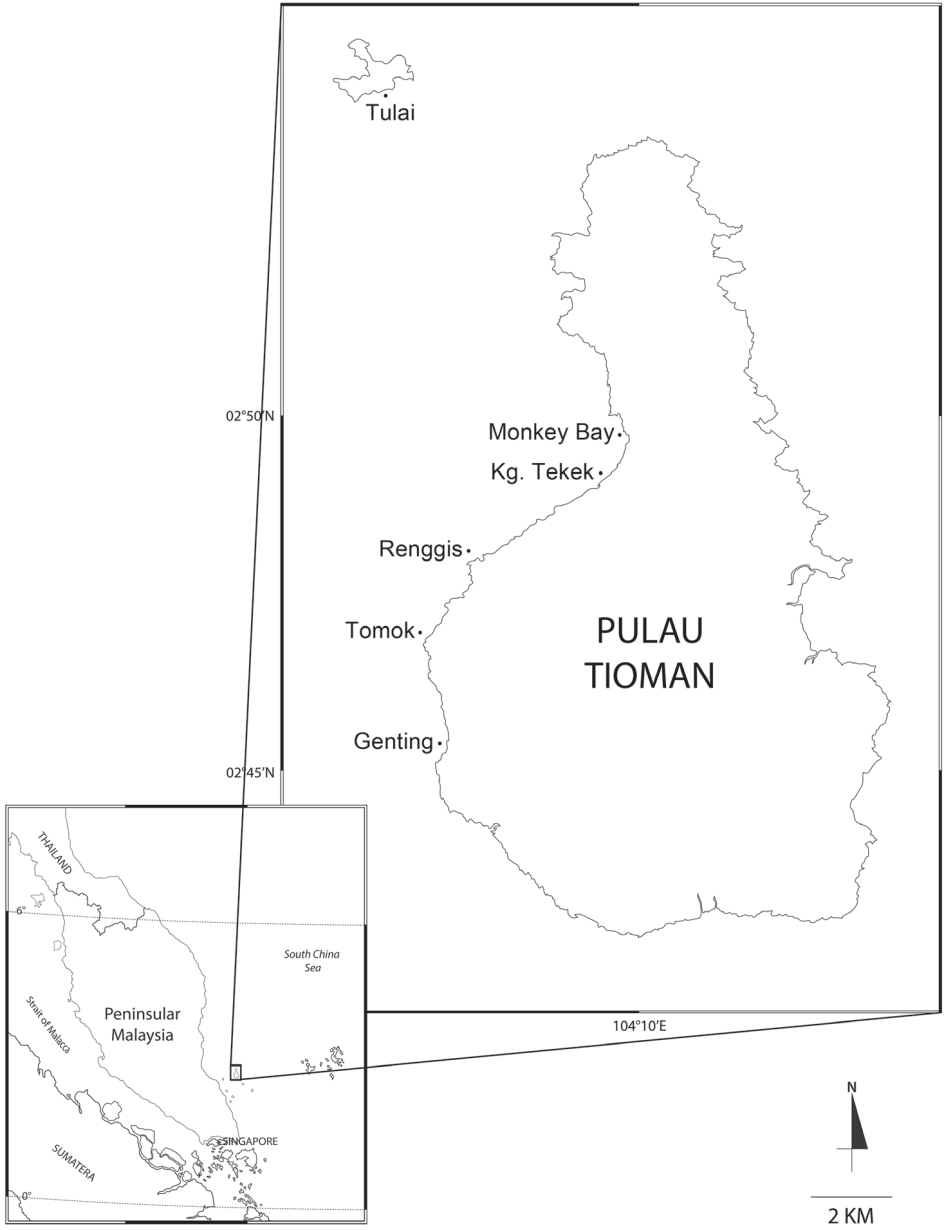
Study area with localities of sampling stations.

The material for the present study came from the following locations in the waters of Pulau Tioman, Pahang (2°48'22"N, 104°10'13"E): Kampung Tekek, Renggis, Monkey Bay, Tulai, Tomok and Genting (Fig. 1). Samples of sea grasses, macroalgae, coral rubble, live corals and intertidal rocks were mainly obtained by SCUBA; amphipod specimens were collected by formalin-wash method (Myers 1985).

Whole animals were transferred into glycerol and drawn with a camera lucida on an Olympus SZX9 dissecting microscope. The specimens were dissected and appendages and mouthparts mounted onto slides in glycerol and drawn under a Leica DMLB light microscope using a camera lucida. Types have been deposited at Universiti Kebangsaan Malaysia Muzium Zoologi (UKMMZ); and the Australian Museum, Sydney (AM). The following abbreviations are used in the figures. A, antenna; ABD, abdomen; EPIM, epimeron; G, gnathopod; HD, head; L, left; LL, lower lip; LM, lacinia mobilis; MD, mandible; MX, maxilla; MP, maxilliped; P, pereopod; PL, pleopod; PLN, pleonite; R, right; T, telson; U, uropod; UL, upper lip; ♂, male; ♀, female.

## Results

### Ampeliscidae Costa, 1857

#### Ampelisca brevicornis

(Costa, 1853)

http://species-id.net/wiki/Ampelisca_brevicornis

Figure 2

Araneops brevicornis Synonymy: Costa, 1853: 171.
Ampelisca brevicornis (Costa, 1853): Chevreux and Fage 1925: 77–79; Schellenberg 1925: 130–133; Pirlot 1936: 277–278; Schellenberg 1942: 146–147; Reid 1951: 204- 210; Nagata 1959: 265–266; Nagata 1965a: 150–151; Imbach 1967: 55–57, pl. 3; Kaim-Malka 1969: 928–932, 934, 953–958; Karaman 1975: 7–12; Rabindranath 1975: 257–261; Lincoln 1979: 112–113; Ledoyer 1982b: 56, 58–59; Hirayama 1991: 86. *Ampelisca* sp. cf. *brevicornis* (Costa, 1853).
Ampelisca laevigata Liljeborg, 1856: Sars 1895: 169–170, pl. 59.

##### Material.

5 specimens, TIO-15, Renggis, Pulau Tioman, 2°48'35"N, 104°8'6"E, washing mix sea grasses, Azman, B.A.R., Rayida, J., 15 July 1999.

##### Remarks.

*Ampelisca brevicornis* is known to be a cosmopolitan species and has been collected from the soft substrata and water column from the littoral zone to the continental shelf from the waters of the world except for boreal areas (Rabindranath 1975; Lincoln 1979; Hirayama 1983). *Ampelicsa brevicornis* was first recorded from the Mediterranean Sea (Costa 1853) by the name *Araneops brevicornis*; it occurs in waters of variable temperatures from north east Atlantic (Schellenberg 1925), south and west Norway (Sars 1895), Atlantic coast of Europe (Lincoln 1979), north Africa (Kaim-Malka 1969; Reid 1951), Indian Ocean (Rabindranath 1975), Japan (Nagata 1965a; Hirayama 1991), Indonesian archipelago (Pirlot 1936) as well as the neighbouring waters of Vietnam (Imbach 1967). Although several morphological variations in antennae, pleonal epimera and urosome are reported between European (Schellenberg 1925) and African specimens (Reid 1951), the present specimens agree in detail with descriptions by Imbach (1967) and Nagata (1959). Imbach (1967) discussed some of the variations concerning this particular species and refuted the idea of naming the existing ecophenotypes reported by Schellenberg (1925), Reid (1951), Dahl (1945) and Pirlot (1936) as a subspecies due to their insignificant distinctions.

Taking into consideration that *Ampelicsa brevicornis* is one of the most popular inhabitants on a littoral sea bottom and widely distributed on the sea floor of the east coast (South China Sea) and the west coast (Straits of Malacca) of Peninsular Malaysia, and numerous specimens have been meticulously examined, misidentification can be confidently ruled out. The specimens at hand show only minor disparities from Imbach’s by having a broader propodus of pereopod 7, so it is clearly satisfactory to identify the specimens as *Ampelicsa brevicornis*.

**Figure 2.**
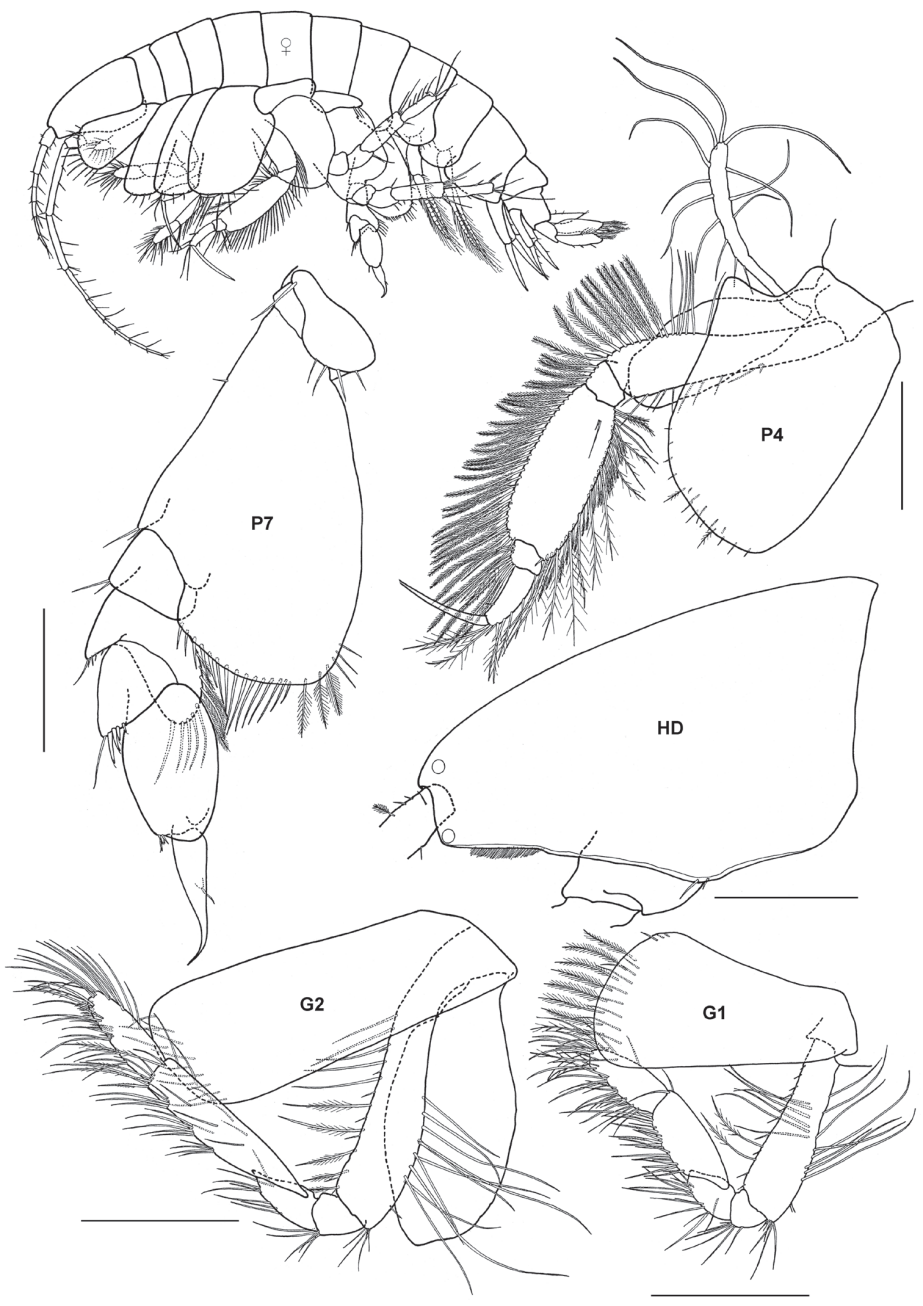
*Ampelisca brevicornis* (Costa), female (UKMMZ-1454), 4.8 mm. Renggis, Pulau Tioman. Scales for **G1, G2, P4, P7** represent 0.5 mm; **HD** scale = 0.2 mm.

### Amphilochidae Boeck, 1871

#### Gitanopsis pusilla

K.H. Barnard, 1916

http://species-id.net/wiki/Gitanopsis_pusilla

Figure 3

Gitanopsis pusilla Synonymy: K.H. Barnard, 1916: 144, pl. 26 (11–12); Griffiths 1973a: 277; Griffiths 1974a: 178; Griffiths 1974b: 224; Griffiths 1974c: 273; Griffiths 1975: 105; Ledoyer 1979a: 17, fig. 3; Ledoyer 1982b: 104–105, fig. 33; Ortiz and Lalana 1997: 106.

##### Material.

5 specimens, TIO-12, Kampung Tekek, Pulau Tioman, 2 2°49'11"N, 104°9'32"E, macroalgae, Azman, B.A.R., Josim, J.J., 11 November 1997; 5 specimens, TIO-15, Renggis, Pulau Tioman, 2°48'35"N, 104°8'6"E, seagrass, Azman, B.A.R., Rayida, J., 15 July 1999.

##### Remarks.

The specimens seem referable to the *Gitanopsis pusilla* without much doubt. In amphilochid amphipods, most species descriptions are based only on females, since the collection of males is rare. The female gnathopods are typical among amphilochids in having a distally dilated propodus, an evenly convex palm and an elongate carpus. The specimens at hand are clearly related to the eastern Pacific species (K.H. Barnard 1916) in bearing the accessory flagellum of antenna 1. Ortiz and Lalana (1997) have reported *Gitanopsis pusilla* and *Gitanopsis antipai* from the neighbouring waters of Bunaken, Indonesia. In their illustration of *Gitanopsis antipai*, the species is set apart from *Gitanopsis pusilla* by the lack of an accessory flagellum, the relatively slender basis of pereopod 7 and the less spinose uropods 1–2.

**Figure 3.**
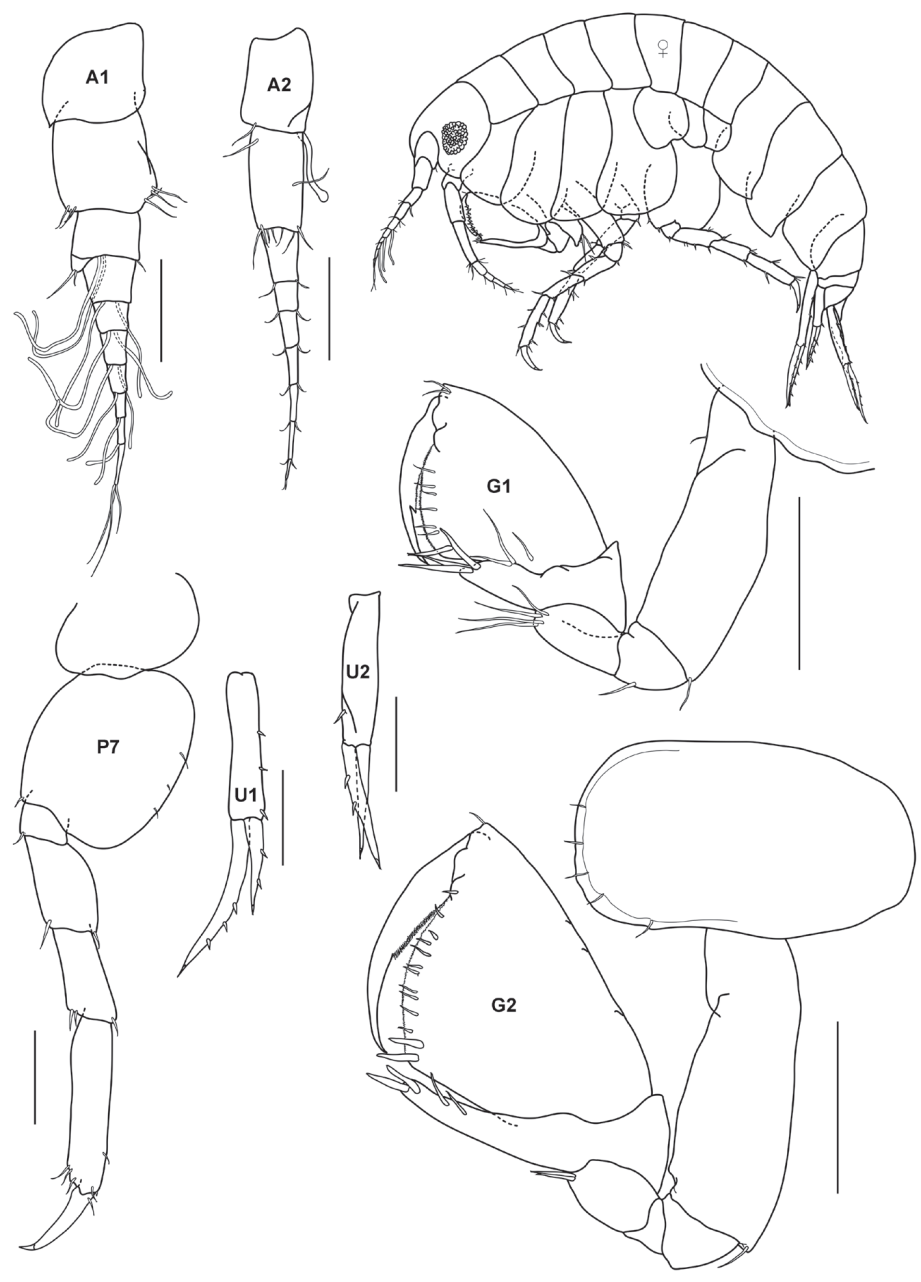
*Gitanopsis pusilla* K.H. Barnard, 1916, female (UKMMZ-1315), 1.7 mm. Kampung Tekek, Pulau Tioman. Scales for **A1, A2, G1, G2, P7, U1, U2** represent 0.1 mm.

### Ampithoidae Stebbing, 1899

#### Cymadusa vadosa

Imbach, 1967

http://species-id.net/wiki/Cymadusa_vadosa

Figure 4

Cymadusa vadosa Synonymy: Imbach, 1967: 89, pl. 32.

##### Material.

3 specimens, TIO 10, Kampung Tekek, Pulau Tioman, 2 2°49'11"N, 104°9'32"E, washing mix macroalgae, Azman, B.A.R., Josim, J.J., 11 November 1997; 4 specimens, TIO 28, Tulai, Pulau Tioman, 2°54'44"N, 104°6'18"E, washing coral rubble, Azman, B.A.R, Kee, A.A., 19 October 2003; 13 specimens, TIO 29, Genting, Pulau Tioman, 2°45'42"N, 104°7'34"E, washing mix macroalgae, Azman, B.A.R., Kee, A.A., 19 October 2003; 5 specimens, TIO 30, Genting, Pulau Tioman, 2°45'42"N, 104°7'34"E, washing mix macroalgae, Azman, B.A.R., Kee, A.A., 20 October 2003.

##### Remarks.

The well-developed sharp spur on the peduncle of uropod 1 distinguishes the present specimens from the genera *Ampithoe* Leach, 1814; *Macropisthopous* K.H. Barnard, 1916; *Amphitholina* Ruffo, 1953; *Psedopleonexes* Conlan, 1982; *Pseudoamphitoides* Ortiz, 1976; *Exampithoe* K.H. Barnard, 1926 and *Melanesius* Ledoyer, 1984. Moreover, the expanded propodus of gnathopod 1 with an oblique palm was found to be a sound character in *Peramphithoe* Conlan & Bousfield, 1982 used in separating *Cymadusa* from *Peramphitoe* (Barnard & Karaman, 1991). *Cymadusa* differs from *Amphithoides* Kossmann, 1880 by having narrow rami of uropod 3 and the absence of telsonic lobes. Moreover *Paragrubia* Chevreux, 1901 differ from *Cymadusa* by having gnathopod 1 larger than gnathopod 2 and in having a multi-articulate accessory flagellum (Poore and Lowry 1997). *Sunamphitoe* Bate, 1857 varies from *Cymadusa* by the absence of a mandibular palp.

Barnard and Karaman (1991) have listed fourteen species of *Cymadusa*, mainly marine and throughout the tropics. Since then another species, *Cymadusa munnu* (Poore & Lowry, 1997) was described from Port Jackson, New South Wales, Australia. Imbach (1967) illustrated *Cymadusa vadosa* on the basis of specimens from south-east Asia region, which he identified with *Cymadusa filosa* (Savigny, 1935), *Cymadusa hawaiiensis* (Schellenberg, 1938), *Cymadusa australis* (K.H. Barnard, 1916), *Cymadusa sardenta* (Oliveira, 1953), *Cymadusa brevidactyla* (Chevreux, 1908), *Cymadusa variata* (Sheard, 1936), *Cymadusa oceanica* (J.L. Barnard, 1955) and *Cymadusa crassicornis* (Costa, 1853), noting some minor differences between them. *Cymadusa vadosa* differs from *Cymadusa filosa*, *Cymadusa hawaiiensis*, *Cymadusa australis*, *Cymadusa compta*, and *Cymadusa microphthalma* by gnathopod 1 with article 5 shorter than article 6.

The Pulau Tioman specimens also appear to be very close to *Cymadusa filosa* in having; (1) accessory flagellum with 1 articulate; (2) peduncle of uropod 1 with strong, acute, distoventral interramal process; (3) uropod 2 with short triangular inter-ramal process.

In all probability, this material from Pulau Tioman is very similar to Imbach’s illustrations in having; (1) article 2 of mandibular palp three fourths as long as article 3; (2) maxilliped extends beyond palp article 2; (3) accessory flagellum with 1 articulate; (4) article 5 shorter than article 6 of gnathopod 1; (5) rami of uropod 3 two thirds as long as peduncle; (6) peduncle of uropod 1 with strong, acute, distoventral interramal process; (7) uropod 2 with short triangular interramal process.

**Figure 4.**
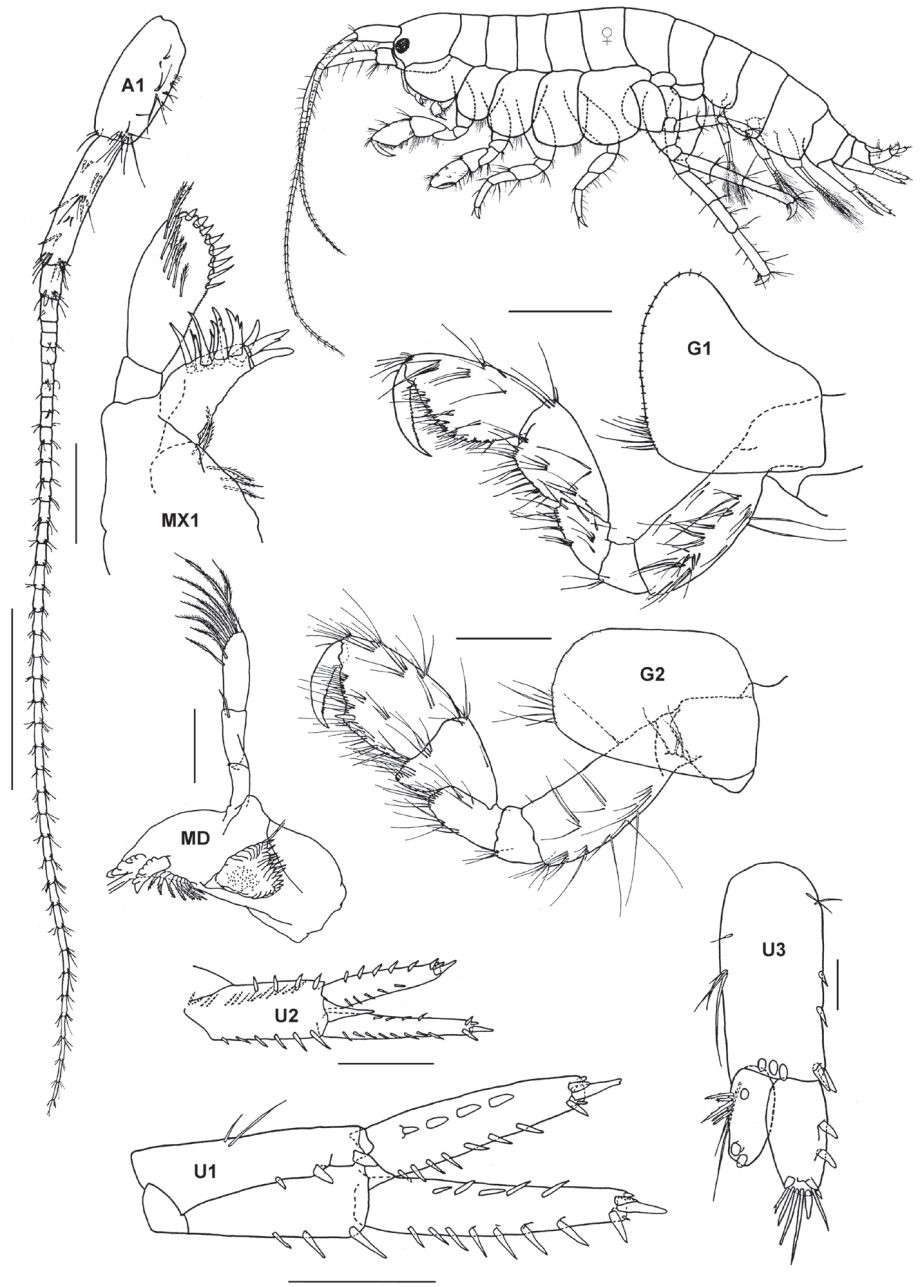
*Cymadusa vadosa* Imbach, 1967, female (UKMMZ-1266), 8.8 mm. Kampung Tekek, Pulau Tioman. Scale for **A1, G1, G2** represents 0.5 mm; **MX1, MX2** scale = 0.25 mm; **U1-U3** scale = 0.2 mm.

### Dexaminidae Leach, 1814

#### Paradexamine setigera

Hirayama, 1984

http://species-id.net/wiki/Paradexamine_setigera

Figure 5

Paradexamine setigera Synonymy: Hirayama, 1984: 225–230; Hirayama 1986: 488.

##### Material.

24 specimens, TIO 10, Kampung Tekek, Pulau Tioman, 2 2°49'11"N, 104°9'32"E, washing mix species of macroalgae, Azman, B.A.R., Josim, J.J., 11 November 1997, UKM I.D. 4891-4898.

##### Remarks.

The Malaysian specimens differ from those described from the waters of Japan only by the lack of the accessory setae of the mandible. At present, this seems inadequate for subspecific distinction due to the enormous resemblance of other characters shared between them. Even though Hirayama (1984) mentioned the closely related *Paradexamine micronesica*, italso lacks the accessory setae of the mandible. Furthermore, the Pulau Tioman specimens show several differences from *Paradexamine micronesica* by having; 1) slenderer basis of pereopod 7; 2) several robust setae on dorsal surface of telson; 3) shorter carpus of male gnathopod 2.

**Figure 5.**
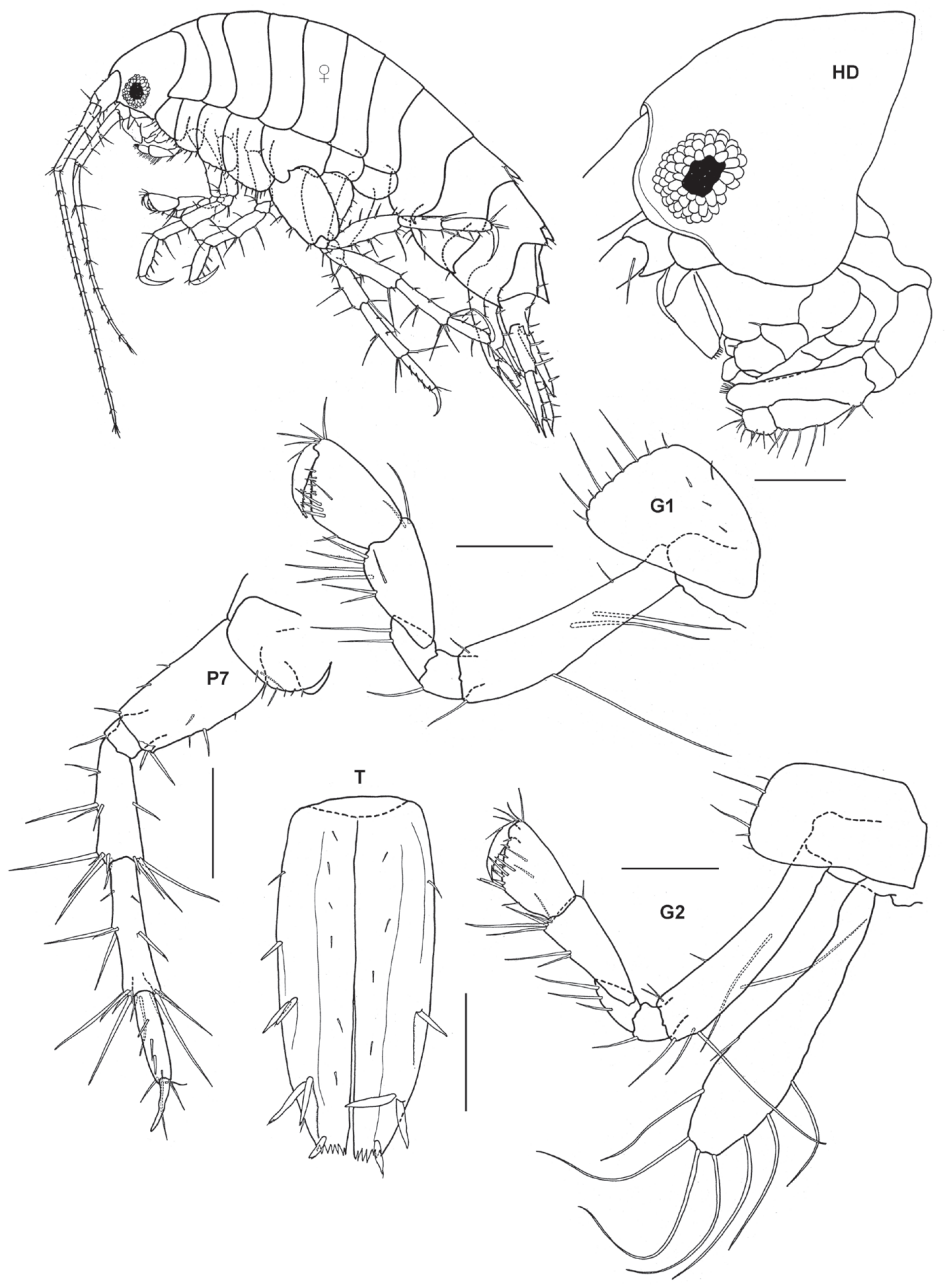
*Paradexamine setigera* Hirayama, 1984, male, (UKMMZ-1259), 2.1 mm. Kampung Tekek, Pulau Tioman. Scales for **G1, G2, HD, T** represent 0.1 mm; **P7** scale = 0.2 mm.

### Ischyroceridae Stebbing, 1899

#### Ericthonius pugnax

(Dana, 1853)

http://species-id.net/wiki/Ericthonius_pugnax

Figure 6

Pyctilus macrodactylus Synonymy: Dana, 1853: 974.
Ericthonius macrodactylus Stebbing, 1906: 672; Walker 1904: 292, fig. 48.
Ericthonius pugnax Stebbing, 1906: 672; Pirlot 1938: 352; Hurley 1954: 445, figs 40–61; Nagata 1960: 179, pl. 179, figs 99–102; Nagata 1965b: 320, fig. 40; Nayar 1967:162; Ledoyer 1969: 179, fig. 1; Hirayama 1985: 52, Ledoyer 1982a: 628, fig. 239; Moore 1988: 727–730, fig. 14; Kim and Kim 1991: 246–247, figs 13–14.

##### Material.

2 specimens, TIO 5, Monkey Bay, Pulau Tioman, 2 2°49'33"N, 104°9'45"E, coral rubble, Azman, Josim, 22 August 1996; 86 specimens, TIO 15, Renggis, Pulau Tioman, 2°48'35"N, 104°8'6"E, seagrass, Azman, Rayida, 15 July 1999, UKM I.D. 5712–5719; 2 specimens, TIO 33, Tomok, Pulau Tioman, 2°47'38"N, 104°7'16"E, live corals (*Porites* sp., *Montipora* sp., *Acropora* sp.), Azman, B.A.R, Kee, A.A., Zuhaimi, S., Maekawa, T., Okashita, T., 22 March 2004.

##### Remarks.

The Pulau Tioman specimens agree well with figures of *Ericthonius pugnax* from Walker (1904), Nagata (1960, 1965c), Ledoyer (1982c) and Moore (1988). This species seems to have a wide-ranging distribution as far as the Arabian Sea. From closer examination of the specimens, the males not only have distally bidentate carpal spur of gnathopod 2 but also a mixture of single dentate carpal spur (see also Nagata 1960; Ledoyer 1969, 1986). As mentioned by Moore (1988) the carpal bidentate spur of ganthopod 2 will eventually be lost in hyperadult males. Unfortunately the stipulation over hyper adult morphological changes has brought several descriptions of new species that actually represented a single species (i.e *Ericthonius macrodactylus*). Prior to this, Ledoyer (1969) synonymized *Ericthonius macrodactylus* as a hyperadult form of *Ericthonius pugnax*. Examination of the Pulau Tioman specimens confirms the presence of this hyper adult morphology: that the basis of pereopod 5 possesses a strongly developed posterodistal lobe. The Pulau Tioman specimens are undoubtedly assigned to *Ericthonius pugnax* and constitute the first record of this species for Peninsular Malaysia.

**Figure 6.**
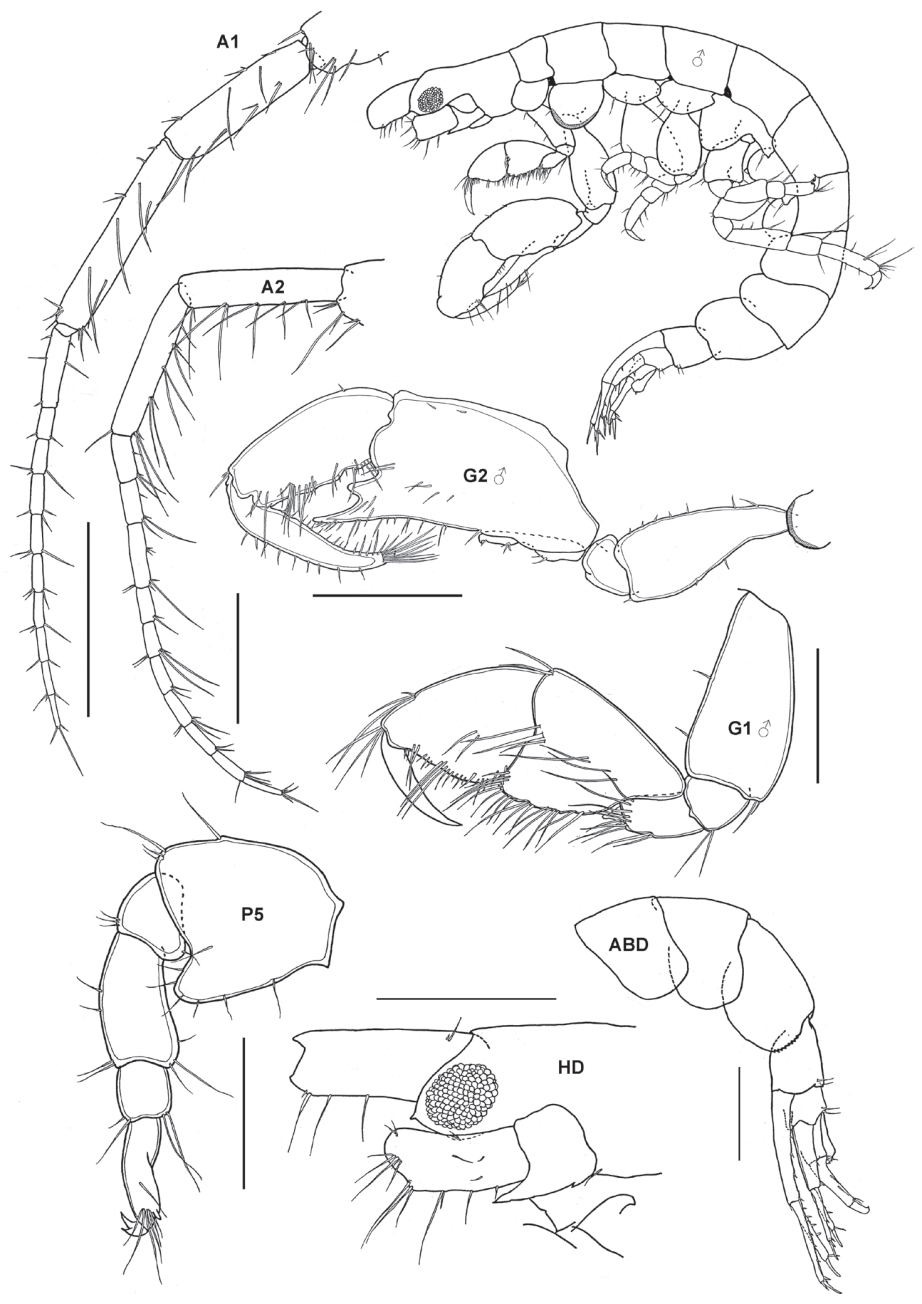
*Ericthonius pugnax* (Dana, 1853), male, (UKMMZ-1136), 3.8 mm. Pulau Tioman, South China Sea. Scale for **G2** ♂ represents 0.6 mm; **A1, A2 HD** and **ABD** scales = 0.5 mm, **G1** ♂ scale = 0.25 mm; **P5** scale = 0.2 mm.

### Leucothoidae Dana, 1852

#### Leucothoe furina

(Savigny, 1816)

http://species-id.net/wiki/Leucothoe_furina

Figure 7

Leucothoe hornelli Synonymy: Walker, 1904: 258–259, pl. 3, fig. 17.
Leucothoe furina (Savigny). Cecchini 1929: 771–773; K.H. Barnard 1931: 120; K.H. Barnard 1937: 152; Pirlot 1936; Ruffo 1938: 156; Nayar 1967: 142, fig. 5d; Imbach 1967: 79, pl. 21; Rabindranath 1967: 387–388, fig. 3; Bussarawich et al. 1984: 4.

##### Material.

4 specimens, TIO 28, Tulai, Pulau Tioman, 2°54'44"N, 104°6'18"E, coral rubble, Azman, B.A.R, Kee, A.A.,19 October 2003; 1 specimen,TIO 31,Tomok, Pulau Tioman, 2°47'38"N, 104°7'16"E, live corals (*Porites* sp., *Montipora* sp., *Acropora* sp.), Azman, B.A.R., Kee, A.A., Zuhaimi, S., Maekawa, T., Okashita, T., 22 March 2004.

##### Remarks.

Referred to many times in the literature from Thailand, this is the first record of *Leucothoe furina* from the intertidal area of the Peninsular Malaysia. The synonymy of this circumtropical species was discussed by Rabindranath (1967). The Pulau Tioman material agrees with the earlier descriptions of Nayar (1967), Imbach (1967) and Rabindranath (1967) with a few variations. The minute accessory flagellum, stouter palp of the mandible, and spinose uropod 1 agrees well with Imbach’s specimen. The gnathopod 2 is unlike Imbach’s illustration, however the nearly smooth palm is also observed in Nayar’s illustration.

**Figure 7.**
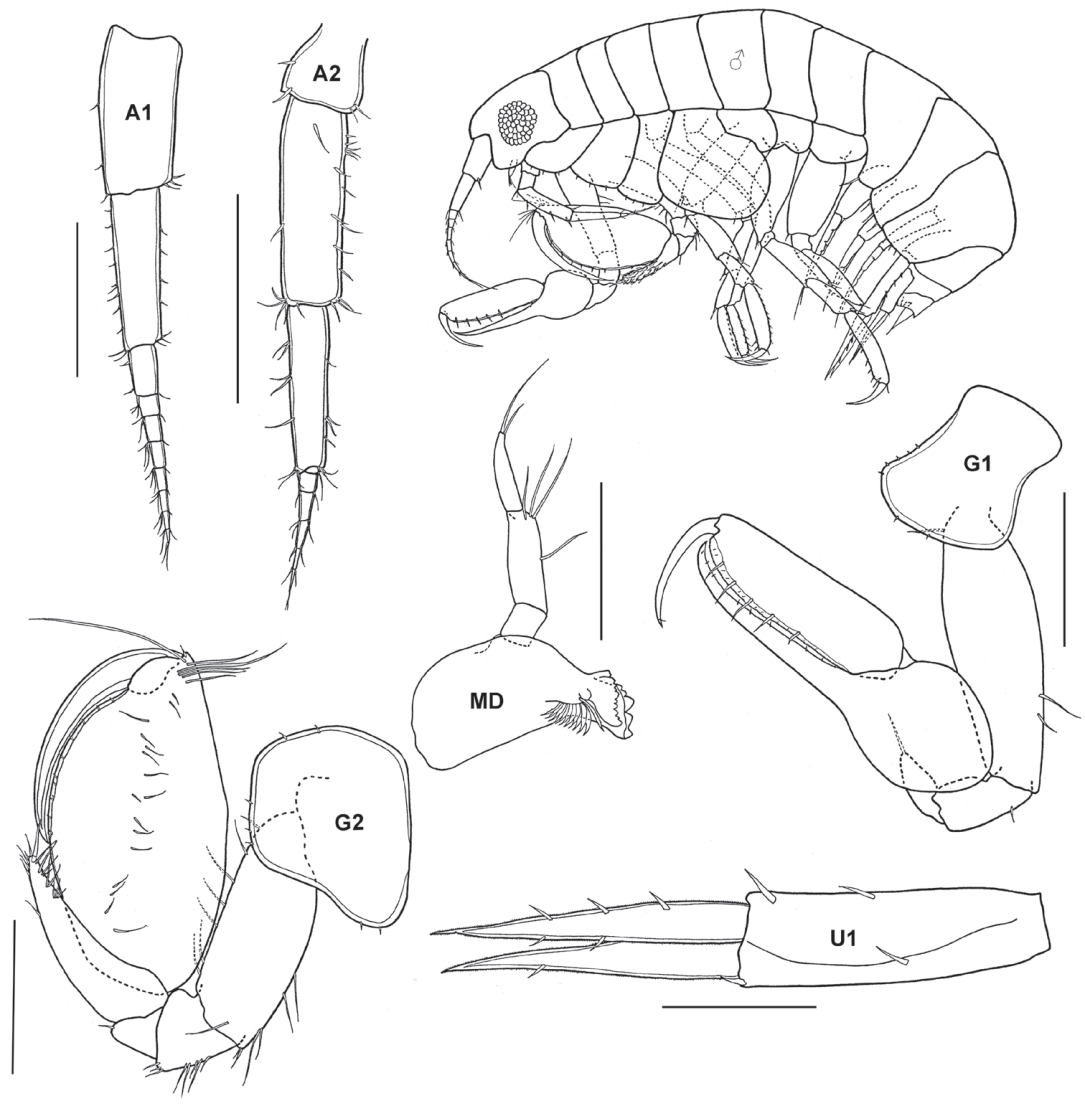
*Leucothoe furina* (Savigny, 1816), male, (UKMMZ-1459), 3.7 mm, Tulai, Pulau Tioman. Scales for **MD** and **U1** represent 0.25 mm; **G1, G2, A1** and **A2** scales = 0.5 mm.

### Liljeborgiidae Stebbing, 1899

#### Liljeborgia japonica

Nagata, 1965b

http://species-id.net/wiki/Liljeborgia_japonica

Figure 8

Liljeborgia japonica Synonymy: Nagata 1965b: 160–164, figs 11–12.

##### Material.

2 specimens, TIO 34, Tomok, Pulau Tioman, 2°47'38"N, 104°7'16"E, live corals (*Porites* sp., *Montipora* sp., *Acropora* sp.), Azman, B.A.R., Kee, A.A., 22 March 2004.

##### Remarks.

The Pulau Tioman material accords well with the description and figures of Nagata (1965b). This is the first record of the species outside Japan.

**Figure 8.**
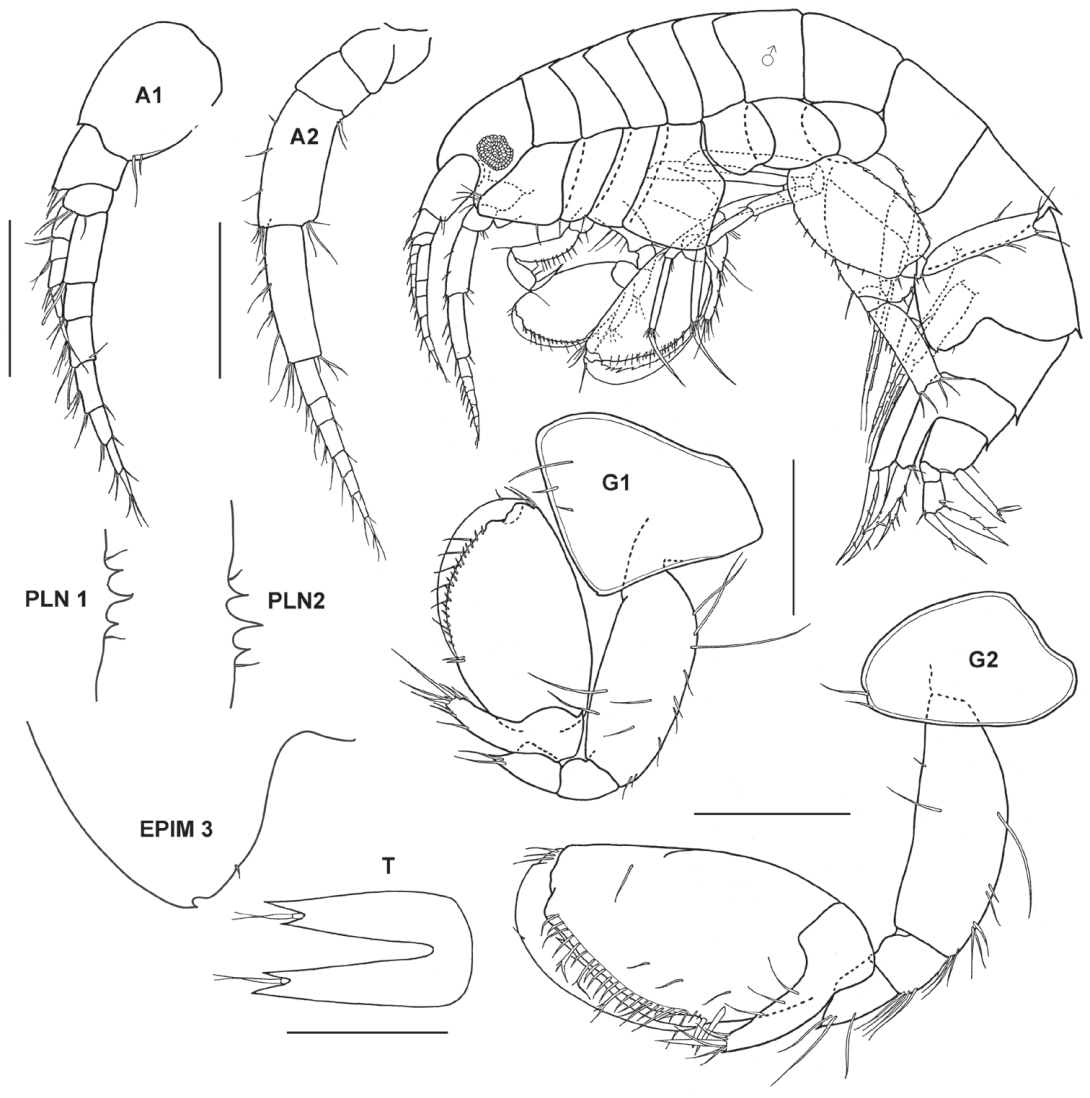
*Liljeborgia japonica* Nagata, 1965, male, (UKMMZ-1224), 3.2 mm. Tomok, Pulau Tioman. Scales for **A1, A2, G1** and **G2** represent 0.25 mm; **T** scale = 0.1 mm.

### Lysianassidae Dana, 1849

#### Microlysias xenokeras

(Stebbing, 1918)

http://species-id.net/wiki/Microlysias_xenokeras

Figure 9

Microlysias xenokeras Synonymy. (Stebbing), 1918: 64, pl. 10; K.H. Barnard 1937: 144; Griffiths 1973b: 293–294, fig. 9; Griffiths 1975: 148–149.

##### Material.

3 specimens, TIO 28, Tulai, Pulau Tioman, 2°54'44"N, 104°6'18"E, coral rubble, Azman, B.A.R., Kee, A.A., 19 October 2003.

##### Remarks.

Griffiths (1975) re-examined this species after discovering an erroneous identification in his earlier publication (see Griffiths 1973a) was based on Barnard’s (1937) *Microlysias indica*. Specimens from Durban Bay, described by Stebbing were the same as Griffiths’s *Microlysias xenokeras*. *Microlysias xenokeras* is the only species in the genus known thus far, and has only been recorded in from the waters of South Africa and Mozambique. It has quite distinctive characters: 1) antenna 1 short and stout, 2) gnathopod 2 minutely chelate, 3) uropod 3 outer ramus 2-articulate, 4) telson with short robust setae dorsally and apically.

**Figure 9.**
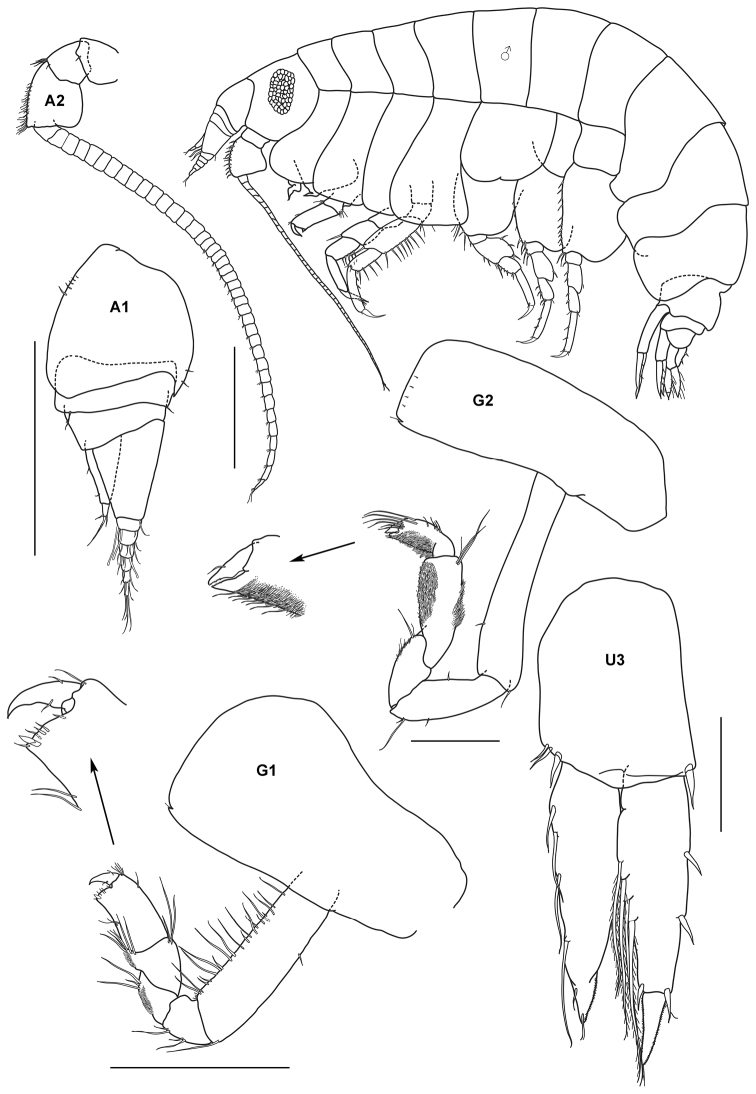
*Microlysias xenokeras* Stebbing, male (UKMMZ-1464), 4.2 mm. Tulai, Pulau Tioman. Scales for **A1, A2, G1** and **G2** represent 0.5 mm; **U3** scale = 0.1 mm.

### Oedicerotidae Liljeborg, 1865

#### Monoculodes muwoni

Jo, 1990

http://species-id.net/wiki/Monoculodes_muwoni

Figure 10

Monoculodes muwoni Synonymy: Jo, 1990: 164–168, figs 5–7.

##### Material.

10 specimens, TIO 3, Monkey Bay, Pulau Tioman, 2 2°49'33"N, 104°9'45"E, coral rubble, Azman, B.A.R., Josim, J.J., 22 August 1996; 1 specimen, TIO 21, Renggis, Pulau Tioman, 2°48'35"N, 104°8'6"E, seagrass, Azman, B.A.R., Rayida, J., 15 July 1999.

##### Remarks.

The Pulau Tioman specimens closely resemble Jo’s (1990) figures described from the Korean peninsula; since then it has not been recorded anywhere else. The short and rather stout rostrum, propodus length of gnathopod 2, parallel sided telson and poorly produced posterodistal corner of coxal plate 4 are several characters unique to this species. However, the identification is not fully satisfactory in the following respects: the long carpal lobes of gnathopods and the double spine rows of the inner ramus of uropod 2. Otherwise, all specimens agree with *Monoculodes muwoni*.

**Figure 10.**
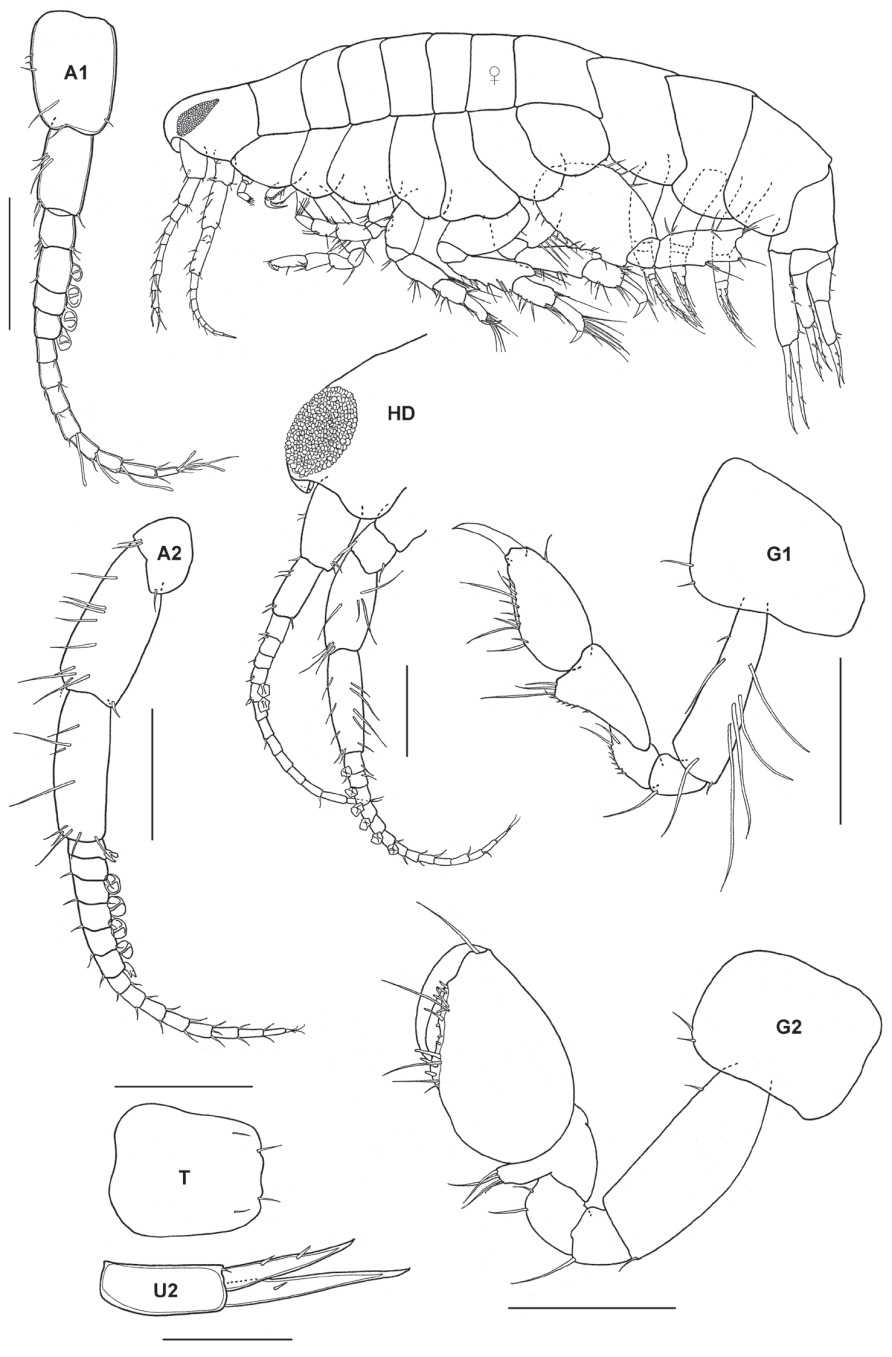
*Monoculodes muwoni* Jo, 1990, female (UKMMZ-1469), 3.3 mm. Monkey Bay, Pulau Tioman. Scales for **A1, A2, HD, G1, G2, U2** and **T** represent 0.25 mm.

### Photidae Boeck, 1871

#### Latigammaropsis atlantica

(Stebbing, 1888)

http://species-id.net/wiki/Latigammaropsis_atlantica

Figure 11

Gammaropsis atlantica Synonymy: Stebbing, 1888: 1101, pl. 114; Ruffo 1969: 43, fig. 13; J.L. Barnard 1970: 174, figs 111–113; Ledoyer 1972: 239, pl. 51–53; Griffiths 1973a: 228; Ledoyer 1979b: 33, figs 13–15; Bussarawich et al. 1984: 4; Myers 1985b: 80, fig. 60; Myers 1995: 52–52, fig. 20; Ortiz and Lalana 1997: 107.
Gammaropsis zeylanicus Walker, 1904: 282, pl. 6 fig. 41; Walker 1909: 339.
Gammaropsis gardineri Walker, 1905: 929, pl. 88 figs 11–14, 16–17.
Gammaropsis atlanticus Stebbing, 1906: 611; Stebbing 1908: 86, pl. 40b; Stebbing 1910: 614, 648; Chilton 1921: 81; Tattersall 1922: 10, pl. 1 figs 17–20; Schellenberg 1926: 375; Hale 1927: 315; Chevreux 1927: 110; Hale 1929: 223, fig. 220. Chevreux 1935: 126; K.H. Barnard 1937: 164; Pirlot 1938: 346; Reid 1951: 258; Pillai 1957: 56, fig. 14; Ruffo 1959: 19; Nayar 1967: 157–158, fig. 13.

##### Material.

5 specimens, TIO 12, Kampung Tekek, Pulau Tioman, 2 2°49'11"N, 104°9'32"E, macroalgae, Azman, B.A.R., Josim, J.J., 11 November 1997.

##### Remarks.

Recently Myers (2009) established the genus *Latigammaropsis* to address J.L. Barnard’s (1970) trepidation on the confusion surrounding the tropical members of the *afra-atlantica* complex, in relation to *Latigammaropsis atlantica* (Stebbing, 1888) and *Latigammaropsis afra* (Stebbing, 1888). The newly proposed *Latigammaropsis* is characterised by the strongly recessed anterodistal margin of the head; lateral cephalic lobes rounded; labrum lacking acute epistome; mandible palp article 3 spatulate; coxae 1–2 without serrations on distal margin; pleon segments lacking spines; uropod 3 peduncle short and broad, rami short and stout; outer ramus blunt-ended with a small second article bearing two fine setae and inner ramus subequal with or shorter than outer ramus, narrowing distally. Which include 16 species namely *Latigammaropsis abbotti* (J.L. Barnard, 1965), *Latigammaropsis afra*, *Latigammaropsis athenae* Myers, 2009, *Latigammaropsis atlantica*, *Latigammaropsis christenseni* (Myers, 1995), *Latigammaropsis dionysus* Myers, 2009, *Latigammaropsis gemina* (Myers, 1995), *Latigammaropsis grandimana* (Ledoyer, 1978), *Latigammaropsis hermes* Myers, 2009, *Latigammaropsis hestia* Myers, 2009, *Latigammaropsis kaumaka* (J.L. Barnard, 1970), *Latigammaropsis pacifica* (Schellenberg, 1938), *Latigammaropsis pali* (J.L. Barnard, 1970), *Latigammaropsis photisimilis* (Ruffo, 1969), *Latigammaropsis planodentata* (Myers, 1995) and *Latigammaropsis togoensis* (Schellenberg, 1925).

The Pulau Tioman specimens undoubtedly represent the tropical members (*afra-atlantica* group) by having an article 2 on the outer ramus of uropod 3 and the inner plate of maxilla 1 has at least 3, often 5+ setae lining the medial margin (Barnard 1970). The presence of the lageniform eye links the specimens at hand with, *Latigammaropsis afra*, *Latigammaropsis athenae* Myers, 2009, *Latigammaropsis atlantica*, *Latigammaropsis photisimilis* (Ruffo, 1969) and *Latigammaropsis hestia* Myers, 2009.

Although the Pulau Tioman specimens are more closely related to *Latigammaropsis gemina*, with the accessory flagellum with 4 articles, inner plate of maxilla 1 with 5+ setae lining the medial margin and occurence of nobs on the urosomal margin. *Latigammaropsis gemina* still does not agree with the specimens at hand in having oval eyes and the telson lacking medial setae.

Nevertheless the specimens at hand are apparently very close to *Latigammaropsis atlantica* in having 1) ocular lobes strongly produced with lageniform eye in hyperadults; 2) antenna 2 shorter than antenna 1; 3) male gnathopod 2 with propodus a little longer than carpus; 4) uropod 1 with strong interramal process, two-thirds length of peduncle; 5) uropod 3, peduncle and outer ramus subequal in length with outer ramus stouter than inner with a small second article and 6) telsonic crests with spines and setae. In addition, the Pulau Tioman specimen appears to be referable to the other known *Latigammaropsis atlantica* that have been recorded from Japan, Bunaken Island, Indonesia, Madras and South Africa.

**Figure 11.**
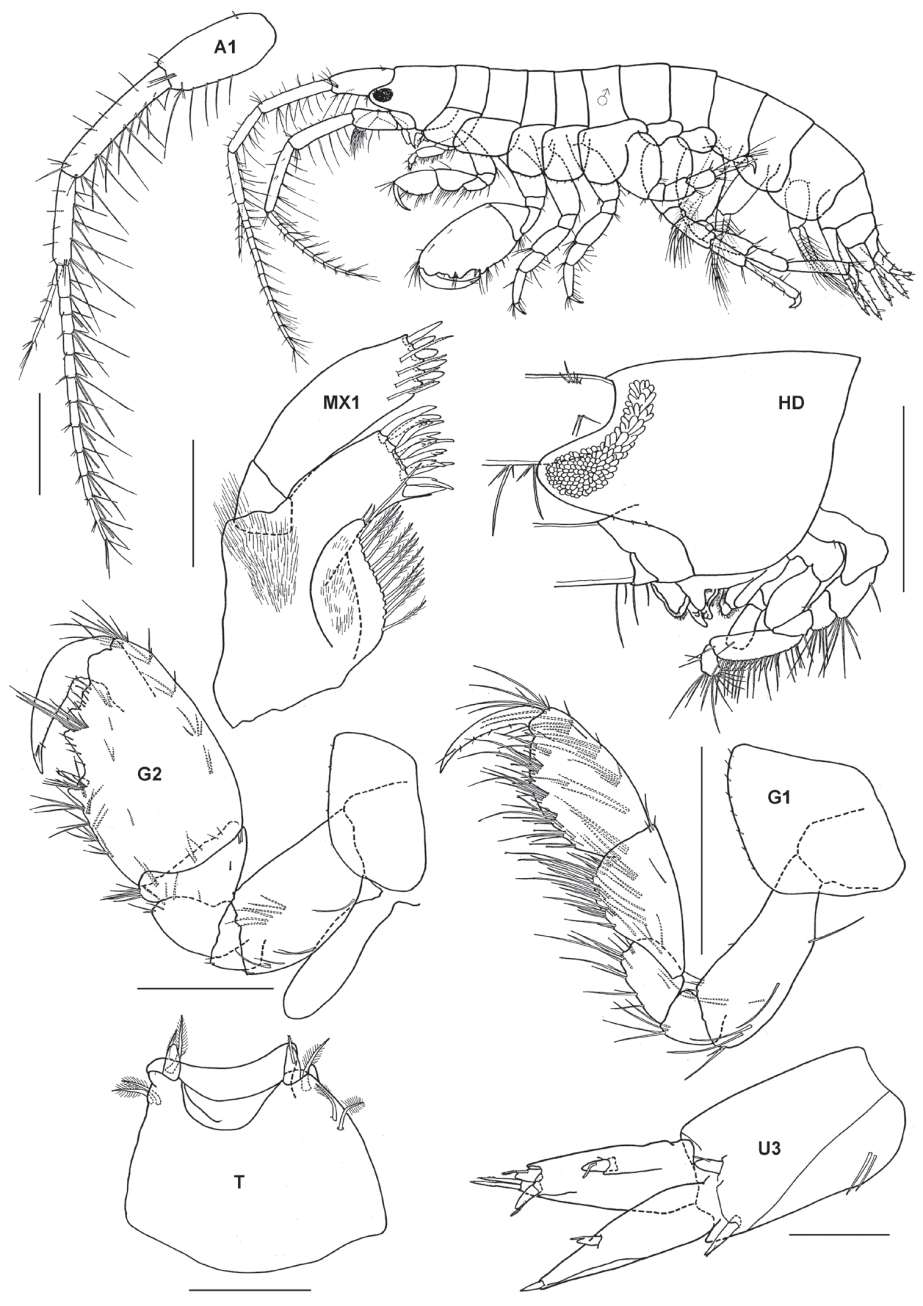
*Latigammaropsis atlantica* (Stebbing), male (UKMMZ-1161), 5.1 mm. Kampung Tekek, Pulau Tioman. Scales for **A1, G1, G2** and **HD** represent 0.5 mm; **T** and **U3** scales = 0.1 mm.

### Family Pontogeniidae Stebbing, 1906
Genus *Tethygeneia* J.L. Barnard, 1972

#### Tethygeneia sunda sp. n.

http://zoobank.org/51D2394A-BF87-49C5-BB06-8F743D836B8A

http://species-id.net/wiki/Tethygeneia_sunda

**Figure 12.**
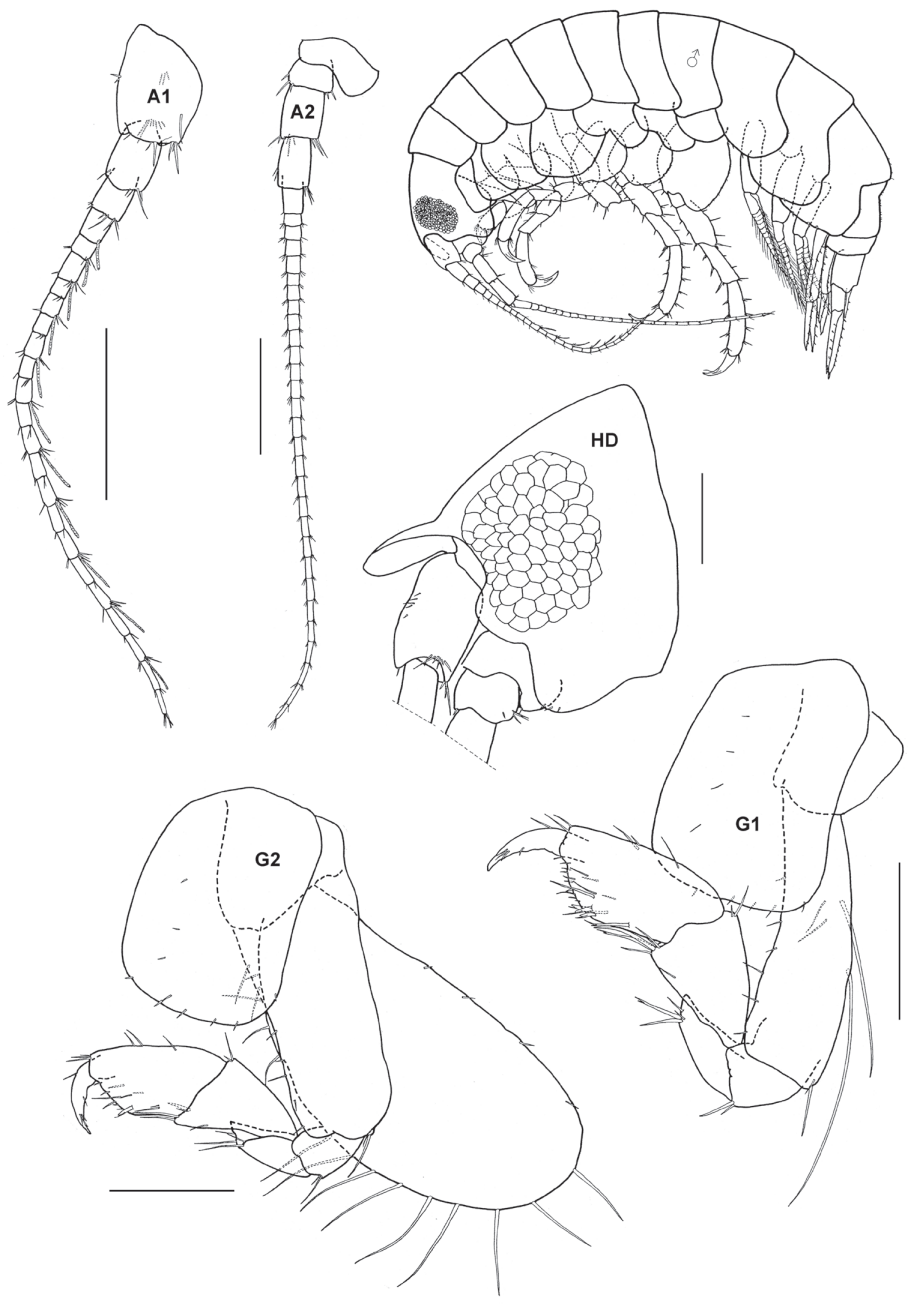
*Tethygeneia sunda* sp. n., holotype, male (UKMMZ-1252), 4.5 mm. Marine Park, Pulau Tioman. Scales for **A1** and **A2** represent 0.5 mm; **G1, G2** and **HD** scales = 0.25 mm.

**Figure 13.**
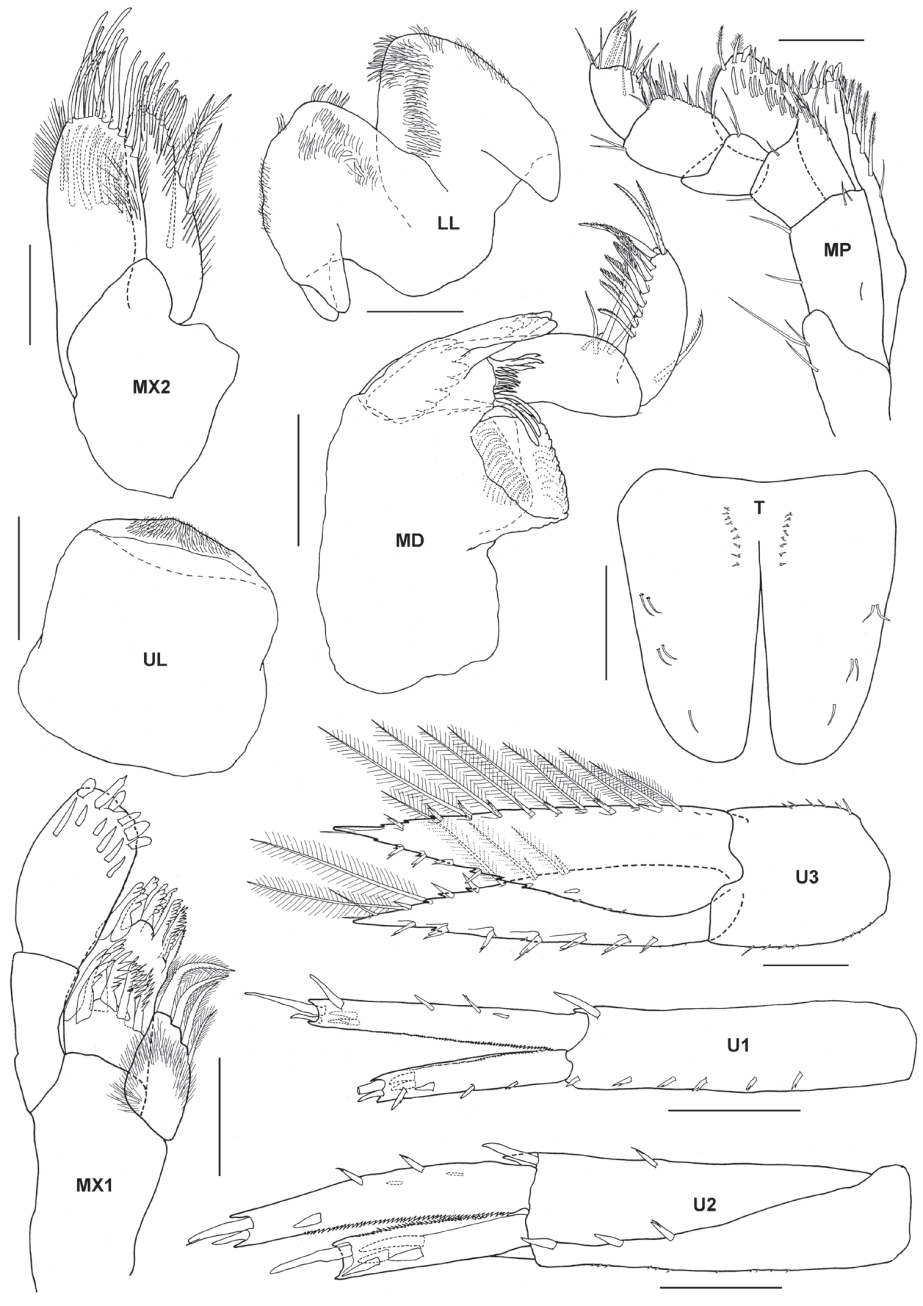
*Tethygeneia sunda* sp. n., holotype, male (UKMMZ-1252), 4.5 mm. Marine Park, Pulau Tioman. Scales for **MX1, MX2, UL, LL, MP, MD, U1–U3** and **T** represent 0.1 mm.

**Figure 14.**
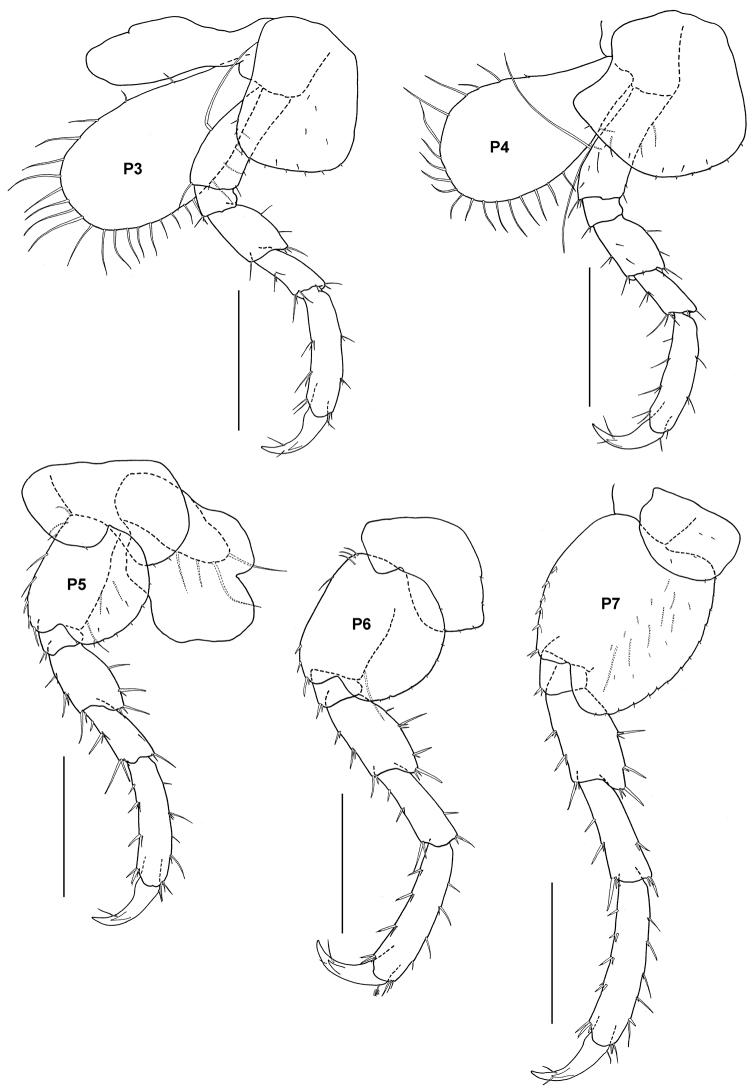
*Tethygeneia sunda* sp. n., holotype, male (UKMMZ-1252), 4.5 mm. Marine Park, Pulau Tioman. Scales for **P3–P7** represent 0.5 mm.

Figures 12 ; 13 ; 14

##### Type material.

Holotype, male, body length 4.5 mm (from tip of rostrum to apex of telson) (Ref: UKMMZ-1252).

##### Type locality.

Marine Park, Pulau Tioman, (2°49'48"N, 104°9'48"E) Peninsular Malaysia; intertidal rocks; coll. Azman, B.A.R., Jusim, J.J., 23 August 2001, UKM I.D. 6687.

##### Additional material examined.

32 specimens, TIO 22, Marine Park, Pulau Tioman, 2°49'48"N, 104°9'48"E, intertidal rocks, Azman, B.A.R., Jusim, J.J., 23 August 2001.

##### Diagnosis.

Rostrum long and linguiform. Accessory flagellum absent. Maxilla 1, palp article 2 stout armed with several short teeth apically. Maxilla 2, outer plate broader than inner plate, both with plumose setae along margin. Lower lip lacking inner lobes. Mandible palp article 2 long. Gnathopod 2 lacking carpal lobe, more slender articles of carpus and propodus. Pereopods 3-4 with pair of stout locking spines. Telson cleft, lacking large spines on apices.

##### Description.

Male: 4.5 mm. Head, rostrum large, long, curved down, apically blunt, lateral cephalic lobe broad, shallow, quadriform, defined below by weak but sharp incision; antero-ventral margin bulbous, rounded. Eye large, occupying more than half of head area.

Antenna 1 only about 70 percent as long as antenna 2; flagellum with about 21-articulate, ornamented with aesthetascs ventrally; accessory flagellum absent. Antenna 2 about half as long as body; gland cone of peduncular article 2 broad, extending beyond of peduncle article 3; flagellum long and thin with about 28 articles.

Mandible, molar triturative, each with ragged seta; lacinia mobilis serrate and clearly distinct; palp with article 2 about twice as broad as article 3, latter slightly falcate. Lower lip lacking inner lobes, mandibular lobes subtruncate and apically fringed with small setae. Maxilliped inner plate with apicolateral spine separated from 2 medioapical spines by gap and hollow; outer plate with facial setules in 1 row and a few scattered; palp article 2 broad. Maxilla 1, palp article 2 stout armed with several short teeth apically. Maxilla 2, outer plate broader than inner plate, both with plumose setae along margin.

Gnathopod 1-2 small, subequal in size to each other, basis scarcely setose posteroventrally; propodus long, thin, sub-rectangular, palm evenly oblique.

Gnathopod 1 carpus sub-triangular, posterior margin short, lobe extended; dactylus not serrate on grasping margin. Gnathopod 2 similar to gnathopod 1. Pereopods 1-7 elongate. Pereopods 3-4 homopodous; merus slightly expanded posterodistally; carpus about ½ the length of propodus. Pereopods 5-7 homopodous; basis expanded roundly. Pereopod 7 similar but longer than pereopod 6; basis more elongate than pereopod 6.

Uropod 1 peduncle spinose on lateral margin, almost 2 times as long as outer ramus, one medium sized robust seta at distal part; outer ramus about 0.7 times as long as inner ramus, apex bifid, armed with several apical spines, one elongate. Uropod 2 extending beyond telson; peduncle subequal in length to inner ramus, spinose, and with one long robust seta at distal end; outer ramus almost 0.6 times as long as inner ramus with several apical robust setae; inner ramus apically bifid with several robust setae along margin. Uropod 3 peduncle short; rami foliaceous, subequal in length, marginally spinose and setose. Telsonflat, broad, cleft more than halfway, apices slightly rounded, broad, smooth, lateral margins of lobes with 2 pairs of partial sets of fine short setae.

##### Remarks.

J.L. Barnard (1972) proposed the genus *Tethygeneia* to group the existing eusiriid amphipods that are limited to a Tethyan distribution, referring to the warm temperate waters of both hemispheres. The key character that clearly differentiates the genus from the other known eusiriids is the long and linguiform rostrum. Although this linguiform nature of the rostrum is also observed in some genera within the family (e.g. *Pontogenia*, *Gondogeneia*), *Tethygeneia* relatively exhibits a stronger form of prolonged rostrum. Since 1991, Barnard and Karaman listed 10 species of *Tethygeneia* that are mostly described from the warm-temperate waters of Australia. *Tethygeneia sunda* sp. n. falls undoubtedly into the genus according to J.L. Barnard’s eusiriid revisional framework (1972). It resembles *Tethygeneia intermedia* (Gurjanova) in lacking a carpal lobe on gnathopod 2, but is rather closely related to *Tethygeneia rostrata* (Gurjanova) and *Tethygeneia longleyi* (Shoemaker) in the other characters. Differing from *Tethygeneia rostrata* in the more slender articles of carpus and propodus of gnathopod 2, in the slightly more produced article 3 of antenna 1, and the more spinose rami of uropod 3. *Tethygeneia longleyi* shares the same form of head, gnathopods and uropods as in *Tethygeneia sunda* sp. n. However some noteworthy differences are observed in the mouthparts, especially the mandible. The length and structure of article 2 of the mandible palp clearly distinguish *Tethygeneia longleyi* from *Tethygeneia sunda* sp. n.

##### Etymology.

The specific designation is derived from the name of the Sunda shelf, which was part of the south-east Asian continent during the Pleistocene.

### Checklist of the recorded amphipods.

**Table 1.** Checklist of the recorded amphipods.

**Family/Subfamily**	**Genus**	**Species**
Ampeliscidae Costa, 1857	*Ampelisca* Kröyer, 1842	1. *Ampelicsa brevicornis* (Costa, 1853)
Amphilochidae Boeck, 1871	*Gitanopsis* Sars, 1895	2. *Gitanopsis pusilla* Barnard, 1916
Ampithoidae Stebbing, 1899	*Cymadusa* Savigny, 1816	3. *Cymadusa vadosa* Imbach, 1967
Dexaminidae Leach, 1814	*Paradexamine* Stebbing, 1899	4. *Paradexamine setigera* Hirayama, 1984
Ischyroceridae Stebbing, 1899	*Ericthonius* Milne-Edwards, 1830	5. *Ericthonius pugnax* (Dana, 1853)
Leucothoidae Dana, 1852	*Leucothoe* Leach, 1814	6. *Leucothoe furina* (Savigny, 1816)
Liljeborgiidae Stebbing, 1899	*Liljeborgia* Bate, 1862	7. *Liljeborgia japonica* Nagata, 1965a
Lysianassidae Dana, 1849		
Tryphosinae Lowry & Stoddart, 1997	*Microlysias* Stebbing, 1918	8. *Microlysias xenokeras* (Stebbing, 1918)
Oedicerotidae Liljeborg, 1865	*Monoculodes* Stimpson, 1853	9. *Monoculodes muwoni* Jo, 1990
Photidae Boeck, 1871	*Latigammaropsis* Myers, 2009	10. *Latigammaropsis atlantica* (Stebbing, 1888)
Pontogeneiidae Stebbing, 1906	*Tethygeneia* J.L. Barnard, 1972	11. *Tethygeneia sunda* sp. n.

## Supplementary Material

XML Treatment for Ampelisca brevicornis

XML Treatment for Gitanopsis pusilla

XML Treatment for Cymadusa vadosa

XML Treatment for Paradexamine setigera

XML Treatment for Ericthonius pugnax

XML Treatment for Leucothoe furina

XML Treatment for Liljeborgia japonica

XML Treatment for Microlysias xenokeras

XML Treatment for Monoculodes muwoni

XML Treatment for Latigammaropsis atlantica

XML Treatment for Tethygeneia sunda
